# A Portable Smartphone-linked Device for Direct, Rapid and Chemical-Free Hemoglobin Assay

**DOI:** 10.1038/s41598-020-65607-8

**Published:** 2020-05-25

**Authors:** Junho Lee, Jaewoo Song, Jun-Ho Choi, Soocheol Kim, Uihan Kim, Van-Thuan Nguyen, Jong-Seok Lee, Chulmin Joo

**Affiliations:** 1 School of Mechanical Engineering, Yonsei University, 50 Yonsei-ro, Seodaemun-gu, Seoul, 120-749 Republic of Korea; 2 Department of Laboratory Medicine, Yonsei University College of Medicine, 50 Yonsei-ro, Seodaemun-gu, Seoul, 120-749 Republic of Korea; 3 School of Integrated Technology & Yonsei Institute of Convergence Technology, Yonsei University, Incheon, 21983 Republic of Korea

**Keywords:** Biotechnology, Health care, Signs and symptoms, Engineering, Optics and photonics

## Abstract

We describe the development and clinical evaluation of an automated smartphone-linked sensor capable of chemical-free, quantitative measurement of hemoglobin concentration ([Hb]) in whole blood samples. We have demonstrated that our sensor could analyze an unprocessed blood specimen with a mean processing time of <8 s and provided the [Hb] results with ~99% accuracy against a reference hematology analyzer with coefficient of variation (CV) of 1.21% measured at [Hb] = 11.2 g/dL. Its diagnostic capability for anemia was evaluated by measuring [Hb] of 142 clinical blood specimens and comparing the results with those from an automated hematology analyzer (ADVIA 2120i, Siemens AG, Germany) and a portable hemoglobinomteter (Hb201+, Hemocue, Sweden). The sensor yielded comparable sensitivities and specificities of 87.50% and 100.00% for males, and 94.44% and 100.00% for females, respectively, for anemic detection. The results suggested that our optical sensor based on the intrinsic photothermal response of Hb molecules and advances in consumer electronics, particularly smartphone capabilities, enables a direct, chemical-free [Hb] assay accessible to people in both developed and developing countries.

## Introduction

Hemoglobin (Hb) constitutes approximately 96% of red blood cells and is responsible for carrying and transporting oxygen to the organs through the circulatory system. The mass concentration of hemoglobin ([Hb]) therefore could be employed as a critical indicator of the oxygen-carrying capacity of blood, and its measurement is routinely performed as a significant and common task in blood assay.

One of the most emblematic blood disorders that can be diagnosed by [Hb] is anemia. In 2015, Global Burden of Disease^1^ reported 2.36 billion individuals to be inflicted with anemia globally, and more than half of them, which corresponds to 1.48 billion individuals, suffered from iron-deficiency anemia. The normal [Hb] range differs by race and age; however, males and females with [Hb] smaller than 13.0 g/dL and 12.0 g/dL, respectively, are generally classified as anemic^2^. Significant modulations in [Hb] might also indicate cardiovascular diseases^3–7^, neurological disorders^8^, dental disorders^9^, excretory disorders^10^, metabolic changes, hepatobiliary diseases, or endocri^,^ne disorders^11^. [Hb] is also an indicator of oxygen homeostasis; thus, [Hb] assay is carried out during or prior to blood transfusion, blood donation^12^ or surgery^13^. A sudden [Hb] change may also be an indicative of a stroke incident in women^14^, sudden loss of blood or regular bleeding^15^.

Representative [Hb] detection methods generally employ chemicals for hemolysis and subsequent generation of hemoglobin-related complexes are detected by colorimetric detectors. The exemplary methods involve the generation of hemiglobincyanide^16^, azide-methemoglobin^17^, and sodium lauryl sulfate-methemoglobin^18^. Clinical grade hematology analyzers and portable hemoglobinometers are mostly based on such protocols. While these methods enable high-accuracy and high-precision [Hb] measurement, some of the employed chemicals are hazardous to human and environment, and therefore the sensors are typically operated by trained operators in controlled sites such as clinical labs. Hematology analyzers enable comprehensive blood assays, but the instruments are expensive, laboratory-based, and require a large volume of blood (>150 µL). On the other hand, portable point-of-care (POC) hemoglobinometers such as Hb201 + (Hemocue, Sweden) and HemoControl (EKF Diagnostics, UK) are relatively inexpensive and simple to operate. Yet, they require long operation time (~15–60 s), toxic chemicals such as potassium cyanide, and large blood volume (>10 µL).

Several chemical-free [Hb] detection technologies have recently been developed. The [Hb] detector based on WHO hemoglobin color scale (HCS) is simple and inexpensive, but its measurement is not quantitative and accurate, and also requires large volume of blood (20 µL)^19^. An automated Hb sensor based on HCS has been introduced capable of operating on a smartphone^20^. However, its operation requires chemicals for reliable operation. Several [Hb] sensing methods that exploit intrinsic physical properties of Hb have been developed. The iron oxide inside hemoglobin exhibits high absorbance at 520–540 nm, and therefore it generates heat under the 532-nm light illumination^21–24^. The resultant temperature increase can be quantitatively measured and converted into [Hb] through electronic and optical transducers. The micro-patterned resistive temperature sensors^25^ have demonstrated high-accuracy detection of [Hb]. However, each measurement requires a dedicated detection chip patterned with temperature sensors and electrodes, which altogether may increase the cost per assay. Several optical methods have been demonstrated. These methods utilize the modulation of refractive index (RI) of blood samples due to photo-thermal (PT) response of Hb molecules under the PT excitation light source. Spectral-domain optical coherence reflectometer demonstrated highly accurate [Hb] detection, but its optical interferometric configuration may pose some issues and challenges in terms of stability and miniaturization^26,27^. Photo-thermal angular scattering (PTAS) also exploits high sensitivity of angular scattering spectroscopy to photo-thermal RI modulations under the illumination of PT light^28^. Compared to others, this method is highly attractive in that it is rapid and requires extremely small volume of blood (<1 µL), which significantly reduces user pains and potential phlebotomy contamination^29^.

Here, we revisit PTAS technology and present a portable, smartphone-linked [Hb] sensor capable of high-accuracy [Hb] assay. We transformed PTAS into a portable and light-weight sensor that can be built with inexpensive consumer electronic devices and operated with any mobile device, such as a smartphone. The sensor, termed mobile-PTAS (“m-PTAS”), features 58 × 68 × 156 mm^3^ in size, and enables chemical-free [Hb] assay with a blood volume of <150 nL. Therefore, it is user- and environment-friendly, and its operating cost is estimated to be <0.20 USD. The m-PTAS sensor was calibrated with reference to a clinical grade hematology analyzer (ADVIA 2120i, Siemens AG, Germany) by using 16 fresh blood samples from healthy volunteers and patients. We then assessed its relative accuracy and precision and compared the results with those of a hematology analyzer (ADVIA 2120i, Siemens AG, Germany). The results were further compared against those from a representative hemoglobinometer (Hb201+, Hemocue, Sweden) to assess its reliability as a portable POC hemoglobinometer. Diagnostic capability of anemic bloods was also evaluated and compared against the results of reference detectors.

## Results

### m-PTAS module

**Figure 1:**
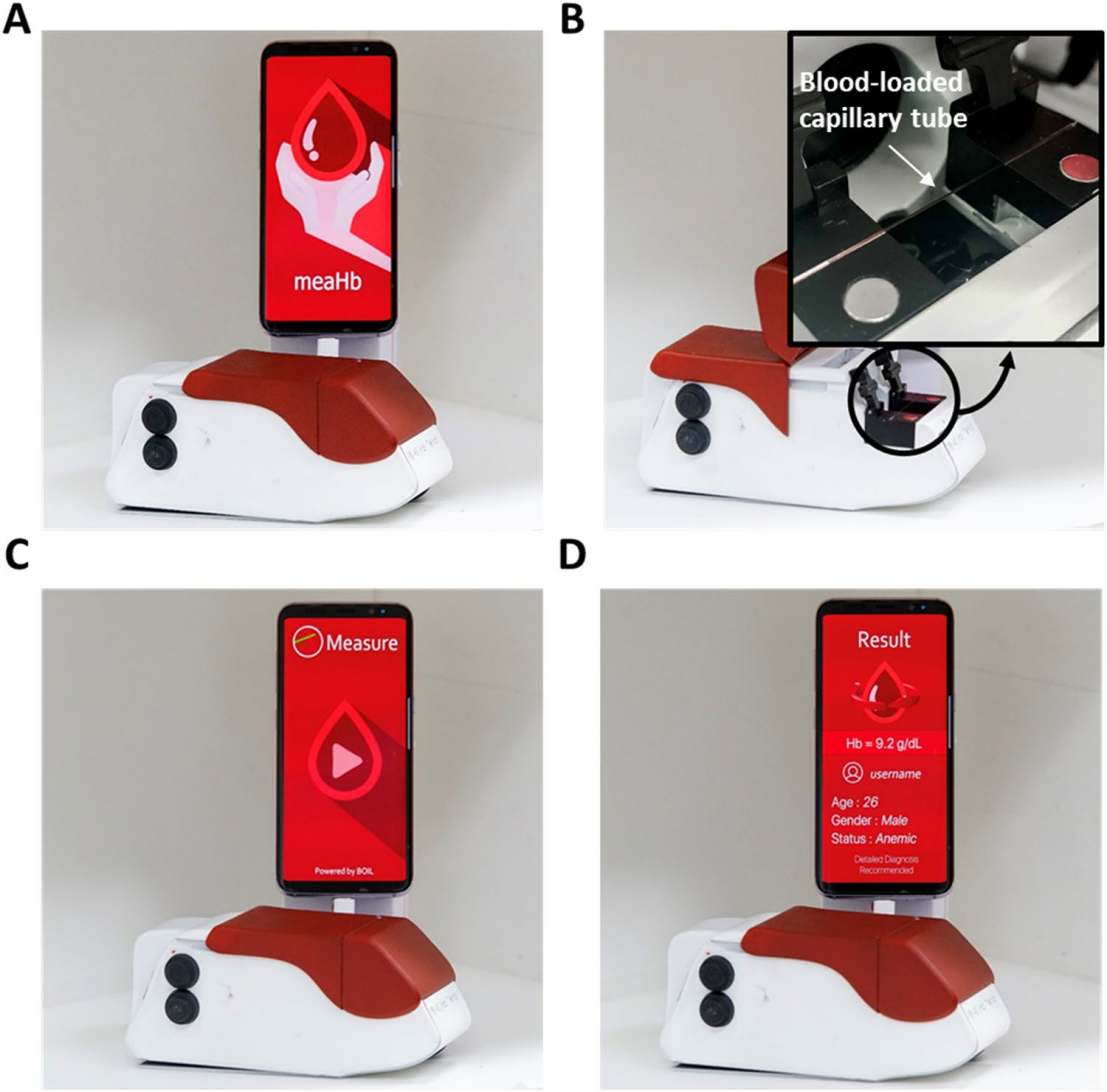
m-PTAS module. **(A)** m-PTAS sensor. **(B)** A user places a blood-loaded capillary tube into a slot in the m-PTAS module. Shown in inset is the magnified view of the capillary tube positioned inside the device. **(C,D)** The device lid is closed, and a dedicated smartphone application (“meaHb”) computes and displays [Hb] on a smartphone.

The m-PTAS module was developed to perform direct, rapid and chemical-free [Hb] assay on a smartphone platform (Fig. 1A). The m-PTAS had dimensions of 52 × 66 × 156 mm^3^ and weighed ~250 g. For [Hb] assay, blood is loaded into a capillary tube via capillary action. The required volume for measurement is approximately measured to be <150 nL. The module is connected to a smartphone and the blood-loaded capillary tube is positioned into the V-mount inside the module (Fig. 1B). The lid of the module is closed, and the module is switched on. The PTAS App, named as ‘meaHb’, is then run using the smartphone (Fig. 1C,D). The smartphone application was developed for operation in Android OS. However, it should be noted that the application can be readily implemented on iOS platform. The application acquires the time-lapse images of scattering patterns under illumination of probe and PT lights for 5 s, and computes and displays the [Hb] of the blood sample (Methods and materials for details). Operation of m-PTAS module is presented in Supplementary Movie S1.

### m-PTAS sensor calibration

**Figure 2:**
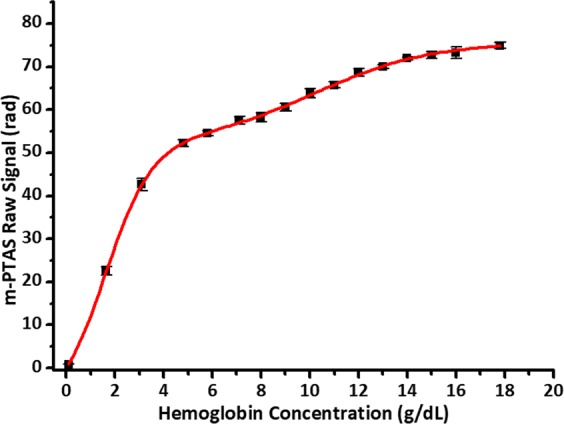
m-PTAS sensor calibration curve. The m-PTAS sensor outputs were calibrated against [Hb] measured by a reference hematology analyzer (ADVIA 2120i, Siemens AG, Germany). Sixteen blood samples of [Hb] ranging from 0.1 g/dL to 17.8 g/dL were analyzed (see Table S1 for detailed information). Each sample was measured ten times, and the error bar denotes 95% confidence interval (CI). The measurement was fitted with a Hill equation (R^2^ > 0.99). Curve fitting constants are given in Table S2.

Calibration of m-PTAS sensor was performed with sixteen whole blood specimens of various [Hb] (0.1 g/dL–17.8 g/dL). Fourteen blood samples ([Hb] from 4.8 g/dL to 17.8 g/dL and plasma) were directly obtained from a clinical laboratory, and two samples of low [Hb] ([Hb] = 1.65 g/dL and 3.1 g/dL) were created by diluting a 6.2 g/dL blood sample with autoplasma. [Hb] of sixteen specimens and their corresponding m-PTAS sensor signals are listed in Supplementary Table S1. The m-PTAS measurements were carried out ten times for each sample, and the outputs were compared against the [Hb] values from a reference hematology analyzer that was calibrated with dedicated calibrators (ADVIA 2120i, Siemens AG, Germany). Figure 2 presents the calibration curve.

As can be noted, m-PTAS sensor exhibited a nonlinear response as a function of [Hb], which agrees to the theoretical estimation made by Gemert *et al*.^30^ Such behavior in the calibration data has been successfully fitted with a Hill equation^27,31^. We performed a curve fitting of m-PTAS calibration results with a Hill equation (R^2^ > 0.99)^32^. The fitting function and its coefficients are listed in Supplementary Table S2. The error bar denotes a 95% confidence interval (CI).

### m-PTAS detection limit

Detection limit of m-PTAS sensor was examined under the guideline EP17, Protocols for Determination of Limits of Detection and Limits of Quantitation published by Clinical Laboratory Standards Institute (CLSI)^33,34^ to obtain limit of blank (LoB), limit of detection (LoD), and limit of quantitation (LoQ) (Table 1). For this evaluation, a blood sample of [Hb] = 9.5 g/dL and autoplasma were used to produce blank and blood samples of low Hb concentrations.

**Table 1:** m-PTAS sensor detection performance.

Analysis Category	Specimen Type	Specimen[Hb] (g/dL)	m-PTAS Sensor				Limit of Blank (g/dL)
			Mean (rad)		SD (rad)	Measurement	
Limit of Blank	Plasma	0.0000	0.0469		0.2811	60	0.0039
**Analysis Category**	**Specimen Type**	**Specimen[Hb] (g/dL)**	**m-PTAS Sensor**				
			**Mean (g/dL)**		**SD (g/dL)**	**Measurement**	**Limit of Detection (g/dL)**
Limit of Detection	Diluted Blood Specimen	0.0316	0.0371		0.0263	60	0.0471
**Analysis Category**	**Specimen Type**	**Specimen[Hb] (g/dL)**	**m-PTAS Sensor**				
			**Mean (g/dL)**	**SD (g/dL)**	**CV (%)**	**Measurement**	**Limit of Quantitation (g/dL)**
Limit of Quantitation	Diluted Blood Specimen	0.0475	0.0492	0.0205	41.74	10	0.1069
		0.0950	0.0877	0.0237	27.04	10	
		0.1188	0.1229	0.0201	16.36	10	
		0.1900	0.1872	0.0224	11.98	10	
		0.2375	0.2529	0.0265	10.49	10	

Detection performance of m-PTAS sensor was quantified based on EP17 guidelines. Diluted blood samples with low [Hb] for LoB and LoQ evaluation were created using the blood sample with [Hb] = 9.50 g/dL and autoplasma.

For LoB quantification, we employed the plasma that was obtained by centrifuging 9.5 g/dL blood sample as the blank. We performed sixty m-PTAS measurements of the samples and the arithmetic mean of the sensor outputs was obtained as LoB = 0.0039 g/dL. In order to quantify LoD, a blood sample of [Hb] = 0.03 g/dL (measured by ADVIA 2010i analyzer) was created by diluting 9.5 g/dL blood with autoplasma, and its [Hb] was measured sixty times by the m-PTAS sensor. We computed the standard deviation (SD) of the measurements, and then evaluated LoD as LoD = LoB + 1.645×SD = 0.0471 g/dL, as instructed by EP 17. Blood samples of five different Hb concentrations were also prepared. The m-PTAS measurements were performed ten times for each sample, and their CVs were evaluated. As guided by EP 17, the measurement results of blood samples of [Hb] = 0.095 g/dL and 0.1188 g/dL were utilized to compute LoQ, as their mean CV value was closest to 20%. The LoQ was then computed as the arithmetic mean of two measurements, yielding LoQ = 0.1069 g/dL.

### m-PTAS precision

We quantified [Hb] measurement precision of the m-PTAS sensor using two quality control (QC) samples provided by Siemens (ADVIA 120/2120/2120i 3 in 1 TESTpoint Hematology Controls, 10316217 and 10318905, Siemens AG, Germany). The [Hb] values of the QC specimens were provided as 11.2 g/dL and 16.4 g/dL by the manufacturer.

In order to evaluate intra-assay imprecision, we performed repeated measurements of the same specimen on the same day. The inter-assay imprecision was, on the other hand, evaluated by measuring [Hb] of the different sample (but with the same Hb concentration) for five days. The measurements were performed five times per sample. For inter-assay evaluation, we adopted EP15-A2 guides recommended by The Clinical Laboratory Standards Institute (CLSI)^35^. The results are summarized in Table 2. Intra-assay imprecision exhibited coefficient of variation (CV) of 1.21% at [Hb] = 11.2 g/dL with standard deviation (SD) of 0.14 g/dL. At [Hb] = 16.4 g/dL, CV was computed as 1.65% and SD was measured as 0.27 g/dL. For inter-assay imprecision, at [Hb] = 11.2 g/dL, CV was calculated as 2.14% with the measured SD of 0.24 g/dL. At [Hb] = 16.4 g/dL, CV was calculated as 1.76% with the measured SD of 0.29 g/dL.

**Table 2:** m-PTAS intra- and inter-assay imprecisions.

Sample [Hb] (g/dL)	m-PTAS					
	Intra-assay Imprecision (n = 5)			Inter-assay Imprecision (n = 5)		
	Mean (g/dL)	SD (g/dL)	CV (%)	Mean (g/dL)	SD (g/dL)	CV (%)
11.2 g/dL	11.24	0.14	1.21	11.19	0.24	2.14
16.4 g/dL	16.45	0.27	1.65	16.45	0.29	1.76

Quality control (QC) samples from Siemens were used to assess m-PTAS precision.

Note that Hb201+ provides intra-assay CV of 0.71% with SD of 0.11 g/dL at [Hb] = 15.36 g/dL, while ADVIA 2120i offers CV of 0.93% with SD of 0.14 g/dL at [Hb] = 15.0 g/dL. These results indicate that m-PTAS exhibits high reproducibility, comparable to ADVIA 2120i and Hb201+.

### m-PTAS relative accuracy

High-accuracy [Hb] detection capability of m-PTAS was then evaluated by measuring [Hb] of 250 blood samples from clinics and by comparing the results against those of the reference hematology analyzer (ADVIA 2120i, Siemens AG, Germany). The performance of m-PTAS sensor was also compared against that of a representative POCT hemoglobinometer (Hb201+, HemoCue, Sweden). The blood samples exhibited randomly distributed [Hb] ranges of 5.2 g/dL–20.6 g/dL. Detailed information of the blood specimens is provided in Supplementary Table S3.

**Figure 3:**
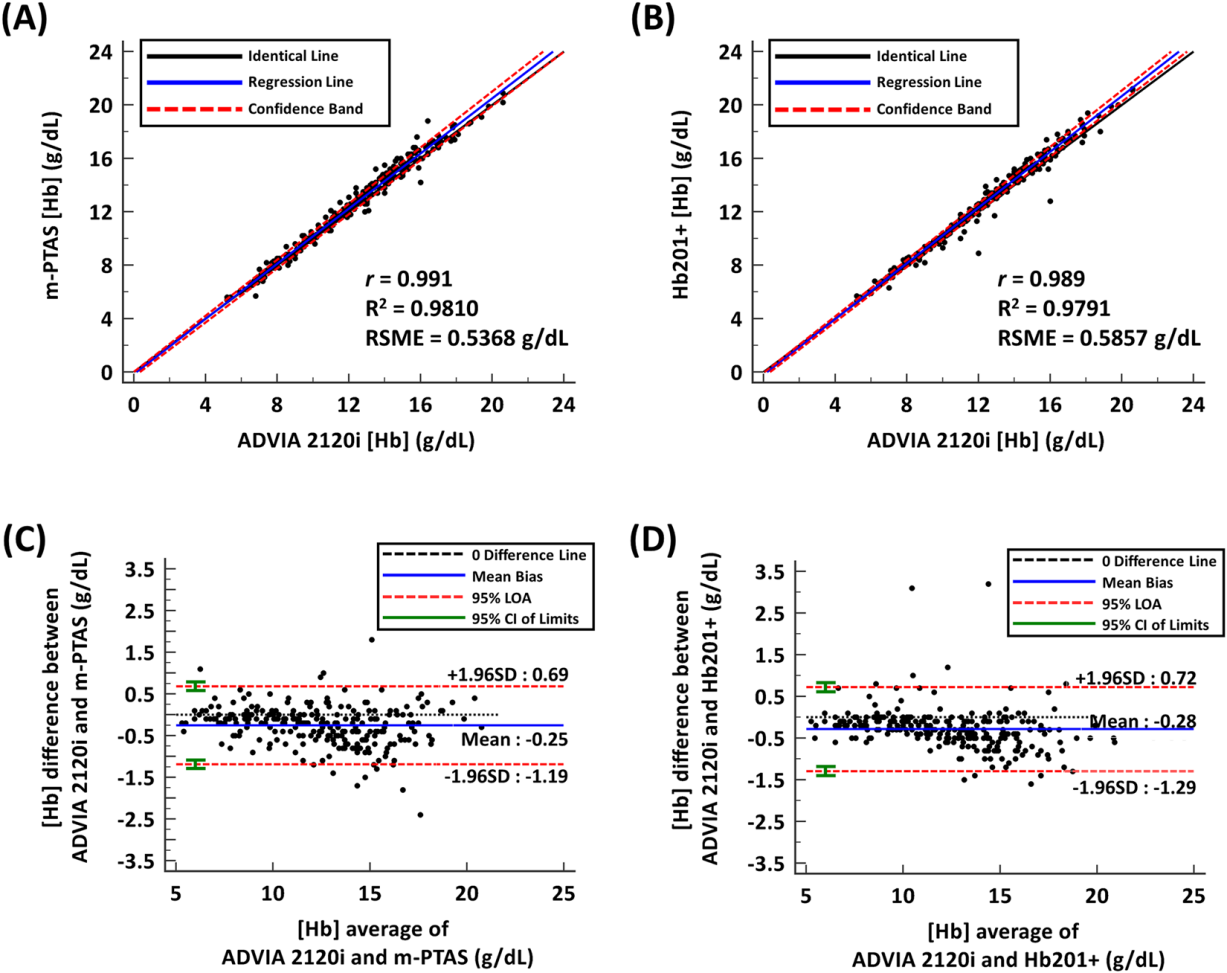
m-PTAS relative accuracy. Passing-Bablok regression analyses for the [Hb] values obtained from **(A)** ADVIA 2120i and m-PTAS, and **(B)** ADVIA 2120i and Hb201+. Solid blue lines represent linear regression lines, and black dash lines represent identical lines. Two red dashed lines represent the confidence band. Bland-Altman analyses for the [Hb] values from **(C)** ADVIA 2120i and m-PTAS, and **(D)** ADVIA 2120i and Hb201+. Black dashed lines represent no difference, and solid blue lines represent mean bias. Two red dashed lines represent 95% limits of agreement. The green error bar represents 95% CI of ± 1.96 SD.

Figure 3A presents Passing-Bablok regression^36^ for [Hb] results from m-PTAS and the ADVIA 2120i analyzer. The blue line indicates a linear regression line, and black solid and red dashed lines represent identical line and 95% confidence band, respectively. The linear regression line is characterized by an intercept of −0.1648 with 95% confidence interval (CI) of −0.3600 to 0.0225 and the slope of 1.0328, with 95% CI ranging from 1.0141 to 1.0500. These results indicate an excellent agreement between the results from ADVIA 2120i and m-PTAS sensor, with a correlation coefficient (r) of 0.991. The R^2^ and root-mean-square error (RMSE) were computed as 0.9810 and 0.5368 g/dL. This result suggests that measurements from the reference hematology analyzer and m-PTAS sensor are in agreement without significant discrepancy. Similar analysis was also performed for Hb201+ against the hematology analyzer (Fig. 3B). The same blood samples listed in Supplementary Table S3 were used for the evaluation. The intercept and slope of the linear regression were found to be −0.2243 and 1.0450, and the corresponding 95% CIs were −0.3824 to −0.0781 and 1.0312 to 1.0588, respectively. The results also indicate a great correspondence between ADVIA 2120i and Hb201 + (r = 0.989, R^2^ = 0.9791 and RMSE = 0.5857 g/dL).

Bland-Altman analysis was further carried out for m-PTAS and Hb201+ against the hematology analyzer, ADVIA 2120i (Fig. 3C,D)^37^. Measurements from m-PTAS and ADVIA 2120i exhibited a mean bias of −0.2496 g/dL with 95% CI ranging from −0.3091 g/dL to −0.1901 g/dL. The standard deviation (SD) was 0.4796 g/dL with limits of agreement (LOAs) ranging from −1.1853 g/dL to 0.6861 g/dL. 95% CI of lower limit ranged from −1.2870 to −1.0835, whereas that for upper limit ranged from 0.5843 to 0.7878. No systematic difference between two measurements was found. Overall, 92.8% of the m-PTAS measurements corresponded to those from ADVIA 2120i within [Hb] difference of 1.0 g/dL. Figure 3D shows the Bland-Altman analysis for the measurements from ADVIA 2120i and Hb201+. The mean bias was measured as −0.2836 g/dL with 95% CI ranging from −0.3476 g/dL to −0.2196 g/dL. SD between the measurements was 0.5102 g/dL and LOA ranged from −1.2900 g/dL to 0.7228 g/dL. 95% CI of lower bound ranged from −1.3994 to −1.1805 and 95% CI of upper bound ranged from 0.6133 to 0.8322. The analysis found no systematic difference or bias, and overall, 91.6% of Hb201+ measurements corresponded to the results from ADVIA 2120i within [Hb] difference of 1.0 g/dL.

### m-PTAS based anemic assay

**Figure 4:**
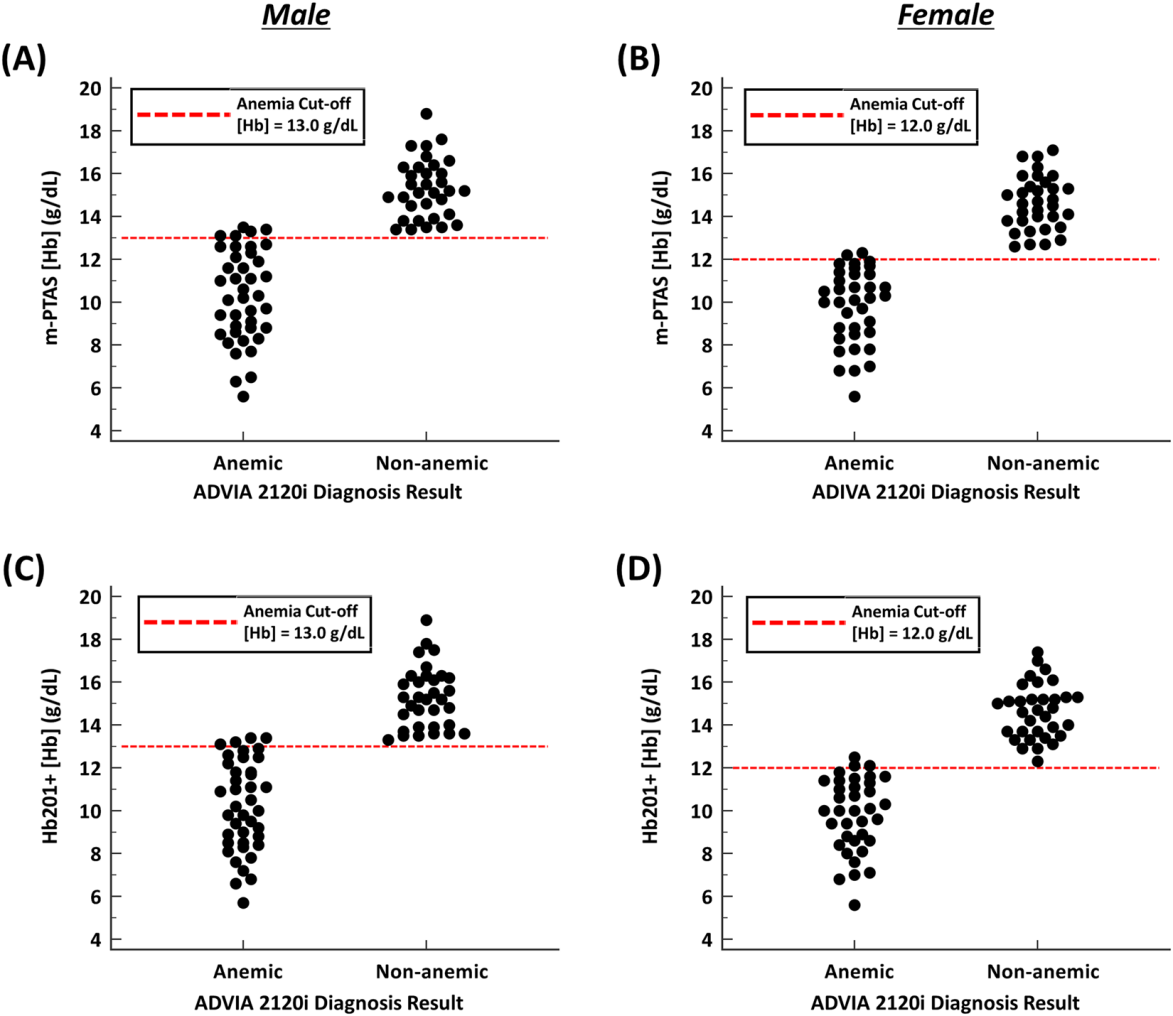
Comparisons of anemia classification results from (**A,B**) ADVIA 2120i and m-PTAS, and (**C,D**) ADVIA 2120i and Hb201+. (**A**,**C)** are the anemia classification results for males, and **(B)** and **(D)** are those for females. The red dashed lines represent clinical anemia cut-offs ([Hb] = 13.0 g/dL for males and [Hb] = 12.0 g/dL for females).

Iron-deficiency anemia is one of the most significant disorders that can be diagnosed by assessing [Hb]. In order to demonstrate clinical significance of m-PTAS, we assessed the detection performance of m-PTAS for anemia. Prior to this evaluation, [Hb] of 142 blood specimens were measured (Supplementary Table S4), and classified into anemic and non-anemic by ADVIA 2120i analyzer with their gender information considered. For anemia diagnosis, clinical cut-offs suggested by World Health Organization (WHO) was employed; anemia is defined by the WHO as a [Hb] below 13.0 g/dL for males and below 12.0 g/dL for females^38^. Figure 4A,B present m-PTAS classification results based on this clinical cut-offs. Red dashed line refers to the clinical cut-off of [Hb] = 13.0 g/dL for males and [Hb] = 12.0 g/dL for females. The m-PTAS diagnosed anemia with 93.15% and 97.10% accuracies for males and females, respectively. The measured sensitivities and specificities were 87.50% and 100.00% for males, and 94.44% and 100.00% for females, respectively. Figure 4C,D show the classification results from Hb201+. Hb201+ classified anemic blood samples with 94.52% and 95.65% accuracies for males and females, respectively. The corresponding sensitivities and specificities were computed as 90.00% and 100.00% for males, and 91.67% and 100.00% for females. Results of statistical analyses are provided in Supplementary Table S5.

## Discussion

The m-PTAS sensor provides rapid, simple, inexpensive, and disposable self-screening and self-monitoring capability for [Hb] and [Hb]-related blood disorders (e.g., anemia), requiring only <150 nL of blood sample. The notable features are (a) chemical-free operation, enabling safe and environment-friendly [Hb] assay; (b) low cost for [Hb] assay (each measurement requires a small and disposable capillary tube, which costs <0.20 USD); (c) small size and portability; and (d) ease of use (a dedicated smartphone app enables users to measure [Hb] themselves).

Our sensor is characterized by high precision with its intra and inter-assay precisions of 1.65% and 1.76% at [Hb] = 16.4 g/dL respectively. A clinical evaluation based on fresh whole blood suggested that [Hb] results from m-PTAS sensor exhibits a high correlation with those of an automated hematology analyzer, ADVIA 2120i (r = 0.991), and yielded excellent sensitivity, specificity and accuracy for anemia detection (sensitivity: 87.50%, specificity: 100.00%, and accuracy: 93.15% for males; sensitivity: 94.44%, specificity: 100.00%, and accuracy: 97.10% for females). Its performance was comparable to that of a representative portable hemoglobinometers, Hb201 + (correlation coefficient: 0.989; sensitivity: 90.00%, specificity: 100.00%, and accuracy: 94.52% for males; sensitivity: 91.67%, specificity: 100.00%, and accuracy: 95.65% for females).

Our smartphone-linked m-PTAS sensor is capable of rapid, automated and quantitative [Hb] analysis, and requires no technical expertise to operate. Therefore, the sensor could be utilized as a convenient [Hb] assay device in local blood centers and for anemia self-screening, especially for those with anemia or other blood-related disorders. In specific, patients susceptible to chronic anemia, including cancer patients undergoing chemotherapy, may utilize this device to monitor their [Hb] at convenience and transmit the results to primary care physicians if their [Hb] levels vary abnormally from the normal baseline. Finally, as our sensor requires an extremely small amount of blood (<150 nL), this device might work with other blood extraction technologies such as laser needle^39^, minimizing blood loss especially for neonates and children.

In terms of m-PTAS implementation, it should be noted that the physical size and cost of m-PTAS module can be further reduced. In our present setup, we employed the complimentary metal-oxide semiconductor (CMOS) image sensor of 1/2.7” type, which features 5.27 × 3.96 mm^2^ in size, to capture a large number of angular scattering patterns on the detector. The measurement of the large number of scattering fringes improves signal to noise ratio and sensitivity of our Fourier-based analysis. Yet, it required the use of optical filters and mirrors of 1” diameter and 0.5” diameter respectively in our case. It should be noted that further miniaturization of m-PTAS sensor can be achieved by use of smaller image sensor and optical elements. In this case, the number of angular scattering fringes may be reduced that can be measured by the detector, but by placing multiple high-power PT micro-LEDs proximal to a blood-loaded capillary, the larger PT response can be induced, thereby improving sensitivity. Instead of multiple optical filters, a customized optical filter directly on top of CMOS sensor may be conceived. These modifications would be a more efficient avenue for future productization. The computation time for [Hb] in the present m-PTAS is determined largely by the central processing unit (CPU) of smartphone, which performs all the computations with images and Fourier analysis. In this demonstration, the computation time was measured to be 2.29 s with a standard deviation of 0.06 s on the platform of Samsung Galaxy S8. Various parallel computational strategies compatible with graphics processing unit may be considered to further improve the computation time.

We demonstrated our m-PTAS sensor in a platform of smartphone to exploit several attractive features and functions provided by the smartphone; it can perform PTAS image processing, conversion of m-PTAS sensor outputs into [Hb] values, display and saving of the [Hb] results, and wireless transfer of the results to remote sites, if necessary. However, it should be noted that the acquired angular scattering images from the m-PTAS module can be transferred to and processed in any types of mobile devices and computers equipped with USB connectivity (e.g., PC, micro PC). The device can also be implemented as the stand-alone [Hb] detector, with dedicated digital signal processing board and display module inside the device. Taken these features together, m-PTAS is expected to be a viable POC hemoglobinometer with demonstrated diagnosing performance.

## Materials and methods

### m-PTAS sensor operation

Optical and electronic architectures of PTAS were re-designed, optimized, and engineered to transform PTAS into a portable, robust and smartphone-linked [Hb] sensor platform, m-PTAS. Its operation is illustrated in Supplementary Fig. S1. A blood-loaded capillary tube is illuminated by a 650-nm probe light, the wavelength of which is outside the Hb absorption range. The incident light is then refracted and scattered by the tube and blood components, and the interference of the scattered light produces a distinct semi-periodic pattern on a detector array. This scattering pattern is found to be highly sensitive to the refractive index of specimen in tube and physical size of the tube^40,41^. The theoretical analysis for this angular scattering pattern can be found elsewhere^42^.

In order to perform chemical-free [Hb] assay via angular light scattering, we exploited intrinsic PT response of Hb molecules. Hb molecules exhibit high absorption at around 520–540 nm, and thus upon the illumination of 532-nm light, Hb molecules inside the capillary tube absorb the incident light energy and convert it into heat^21–24^. The resultant temperature rise decreases the refractive index (RI) of the blood inside the tube^43^, which subsequently shifts the angular light scattering pattern (Supplementary Movie S2). We observed that the amount of the scattering shift is proportional to the amount of hemoglobin in the blood, and thus we quantified the shift of the angular scattering pattern to perform quantitative [Hb] assay. For measurement, we acquired time-lapse images of scattering patterns at a frame rate of 30 Hz for 5 s under the intensity modulated PT light illumination. We employed intensity-modulated illumination for the PT light, as it facilitated measurements with high signal to noise ratio (SNR). The modulation frequency was set to 1 Hz.

We computed m-PTAS sensor output as detailed in Kim *et al*.^28^ Supplementary Fig. S2 outlines the signal processing method. For each image frame, we obtained an averaged scattering pattern by calculating the mean of the pixel values of the image along the vertical direction. Supplementary Figs. S2A and S2B present exemplary images and corresponding averaged scattering patterns before and after PT light illumination. The measured scattering pattern was then Fourier transformed. Since the angular scattering pattern was characterized by a distinct spatial frequency, its Fourier counterpart could readily be located. We measured the phase of the scattering signal in the Fourier domain during the modulated PT light illumination. Supplementary Fig. S2C shows phase fluctuations during the modulated PT illumination. The measured phase fluctuation was then Fourier transformed, and its magnitude at the modulation frequency, termed the m-PTAS sensor signal, was examined (Fig. S2D).

### m-PTAS fabrication

The main frame and opto-mechanical structures of the m-PTAS were designed using SolidWorks (Solidworks 2016, Dassault Systèmes SolidWorks Corp., France) and manufactured with a stereo lithography apparatus (SLA) based 3D printer (KINGS 3035 Pro SLA 3D Printer, Shenzhen Kings 3D Printing Technology, China). The base material was resin except for some add-in structures inside the module. Figure S3 depicts the optical setup inside the module. 650-nm (650MD-1-0618, Besram Technology Inc., China) and 532-nm (532MD-200-3*7 V, Lilly Electronics, China) laser diodes were employed as the probe and PT excitation light sources. The light from the laser diodes (LDs) illuminated and overlapped in a capillary tube (CV2033, CM Scientific, England), that features inner and outer diameters of 200 μm and 330 μm, respectively. Note that a 300-mm long capillary tube costs approximately 1.1 USD, and the required tube length for a reliable Hb assay was 50 mm in our device. Therefore, the 300-mm long tube was cut into 6 pieces, and the cost per each assay was estimated to be 1.1 USD/6 = 0.18 USD (<0.20 USD). We placed a circular aperture (700 μm in diameter) in front of the probe LD to ensure that the probe light is smaller than the PT excitation light. The aperture was 3D printed (Wiiboox One, Measurement Korea, Republic of Korea). The beam spot sizes of the probe and PT light were measured to be 0.57 mm and 0.82 mm on the capillary tube. The intensity of the PT light was 90.5 mW/cm^2^.

The scattering pattern of the 650-nm probe light produced by the capillary tube was captured by a complimentary metal-oxide semiconductor (CMOS) sensor (ELP-USBFHD01M-L21, Ailipu Technology Co., Ltd, China). The sensor is equipped with USB connection, through which the detector is powered, and data are transferred. In our device, the PT laser diode emitted light at 532-nm, 808-nm, and 1064-nm simultaneously. Hence, neutral density (NE30B, Thorlabs, USA), long-pass (FGL610, Thorlabs, USA), and infrared cut-off filters (12.5 mm Diameter IR Cut-Off Filter, Edmund Optics Korea, Republic of Korea) were stacked and inserted in front of the image sensor to ensure detection of only 650-nm probe light without saturation. The module was 52 × 66 × 156 mm^3^ in size. Detailed illustrations of 3D module frame and fully assembled m-PTAS sensor are presented in Supplementary Figure S4.

To achieve PTAS measurement with high SNR, the 532-nm PT laser was modulated at 1 Hz. The modulation was performed by Arduino-nano (SZH-EK025, Jiangsu Yuheng Co., Ltd, China) with pulse-width modulation (PWM) control.

The bill of materials for our m-PTAS implementation is provided in Supplementary Table S6. It should be emphasized that the cost can be further reduced by using high-power micro-LEDs, and smaller image sensor and optical elements.

### Smartphone application

Our application is based on the Android platform, which provides various processing features such as recording sensor data obtained from the external sensor devices connected through USB. In order to perform robust and computationally effective analysis, as described in *m-PTAS sensor operation*, we employed the following strategies and optimizations to our software implementation. The sequential image data were acquired from the CMOS sensor for 5 s, where the libusbcamera library^44^ was adopted. The sensor data acquisition and data processing procedures operated separately, instead of executing in parallel. This prevented the unintended interruption of the data acquisition process due to the computation constraints of the mobile device. Moreover, the multithreaded video decoding was employed to utilize all the CPU cores, and to maximize the computational power, C++ -based implementations were employed in several heavy operations such as image pre-processing.

We deployed our mobile application to a Samsung Galaxy S8 smartphone running on Android 8.0.0 operating system (OS). The device contains a Samsung Exynos 8895 system-on-chip (SoC), which consists of eight CPU cores. In addition, this device supports connection with an external sensor device through its USB 3.0 Type-C port, which provides sufficient data bandwidth to receive images having a resolution of 1920 × 1080 pixels at a frame rate of 30 Hz. Overall computation takes 2.29 s on average with a standard deviation of 0.06 s (Supplementary Table S7).

### Sample Preparation (IRB)

This study was approved by the Institutional Review Board (IRB) of Severance Hospital (Seoul, Republic of Korea), an affiliated hospital of Yonsei University Health System (Approval Number: 1-2016-0037). The clinical blood samples were residual blood samples that had been acquired and processed in clinical labs. The acquisition of written consent forms from the patients was waived for using the remaining blood samples after clinical laboratory tests under the IRB. All experiments were conducted in accordance with principles expressed in the Declaration of Helsinki or other relevant guidelines and regulations. The blood samples were supplemented with tripotassium ethylenediaminetetraacetate (K3EDTA) to prevent coagulation. For experiment, all the samples were obtained on the same day unless specified otherwise, and measured within three days. The samples were stored at room temperature (23 °C).

For m-PTAS calibration, fresh 16 blood specimens of different [Hb] (0.1 g/dL, 4.8 g/dL, 5.8 g/dL, 7.1 g/dL, 8.0 g/dL, 9.0 g/dL, 10.0 g/dL, 11.0 g/dL, 12.0 g/dL, 13.0 g/dL, 14.0 g/dL, 15.0 g/dL, 16.0 g/dL, and 17.8 g/dL) were prepared. The samples of [Hb] = 0.35 g/dL and 1.5 g/dL were prepared by diluting the 6.2 g/dL blood sample with plasma gathered from centrifuged blood sample of [Hb]=13.1 g/dL. Other specimens were directly obtained from the clinical laboratory.

For LoB, LoD and LoQ quantification of m-PTAS sensor, the blank and blood samples of low Hb concentrations were all created by diluting 9.4 g/dL blood with autoplasma. Prior to m-PTAS assay, the [Hb]s of all the samples were measured by ADVIA 2010i analyzer. For precision test, two quality control (QC) samples from Siemens AG (ADVIA 120/2120/2120i 3 in 1 TESTpoint Hematology Controls, 10316217 and 10318905, Siemens AG, Germany) were used. [Hb] value of both samples, 11.2 g/dL and 16.4 g/dL respectively, were specified by the manufacturer.

For evaluation of relative accuracy, a total of 250 clinical blood samples from pediatric and adult patients were gathered, measured, and analyzed. For anemia assay, 142 blood samples with the gender information were gathered. [Hb] was measured by the clinical grade hematology analyzer (ADVIA 2120i, Siemens AG, Germany), a portable hemoglobinometer (Hb201+, Hemocue, Sweden), and m-PTAS. The [Hb] values from the automated hematology analyzer were not revealed to m-PTAS operators to ensure objective evaluation.

## Supplementary information

Supplementary Information.

Supplementary Information 2.

Supplementary Information 3.
