# Supplementary material for: A Portable Smartphone-linked Device for Direct, Rapid and Chemical-Free Hemoglobin Assay
Source: Sci Rep. 2020 May 25;10:8606. doi: 10.1038/s41598-020-65607-8 (PMC7248091; doi:10.1038/s41598-020-65607-8)
Supplement: Supplementary file 3 — Supplementary Information 3. [file 41598_2020_65607_MOESM3_ESM.pdf]

# Supplementary Materials

## A Portable Smartphone-linked Device for Direct, Rapid and Chemical-Free Hemoglobin Assay

Junho Lee[1,a], Jaewoo Song[2,a], Jun-Ho Choi[3], Soocheol Kim[1], Uihan Kim[1], Van-Thuan Nguyen [1], Jong-Seok Lee[3], and Chulmin Joo[1]*

[1]School of Mechanical Engineering, Yonsei University, 50 Yonsei-ro, Seodaemun-gu, Seoul 120-749, Republic of Korea.

[2]Department of Laboratory Medicine, Yonsei University College of Medicine, 50 Yonsei-ro, Seodaemun-gu, Seoul 120-749, Republic of Korea.

[3]School of Integrated Technology & Yonsei Institute of Convergence Technology, Yonsei University, Incheon, 21983, Republic of Korea

[*]To whom correspondence should be addressed: cjoo@yonsei.ac.kr (C. Joo)

[a]These authors contributed equally to this work.

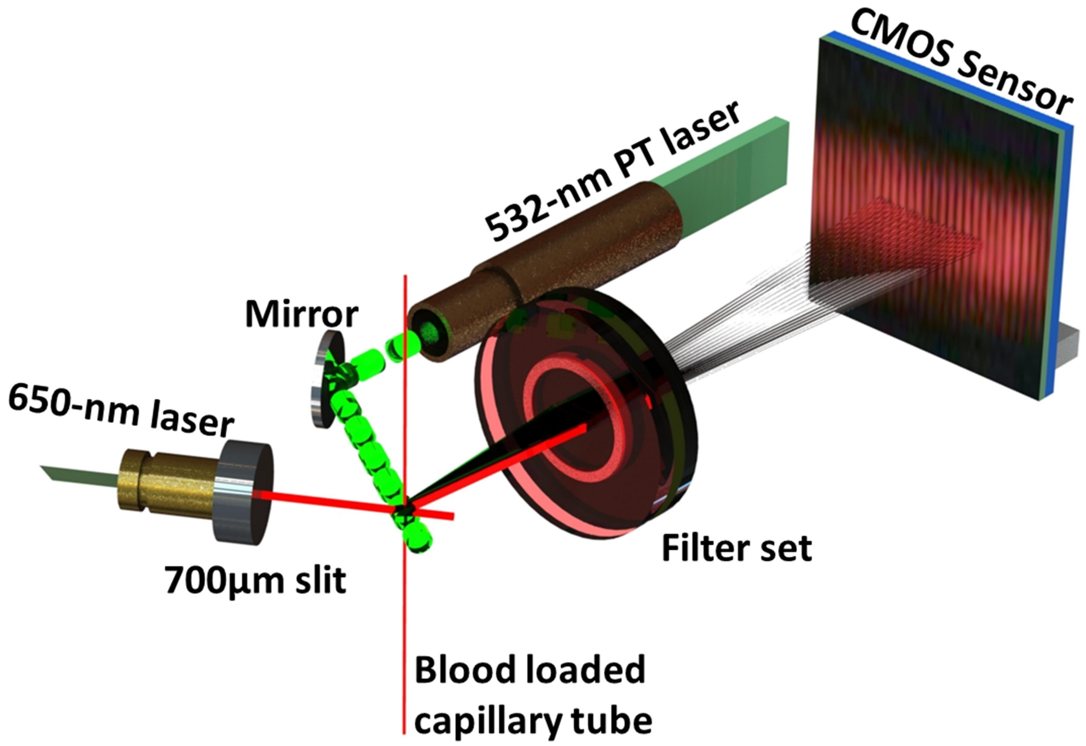


**Fig. S1.** m-PTAS operation. A 650-nm probe light is directed to a blood-containing capillary tube. The interference of the refracted and scattered light from the tube produces a distinct semi-periodic pattern on an image sensor. Under the illumination of 532-nm PT excitation light, where Hb exhibits high absorption, Hb absorbs the light energy and converts it into heat, leading to a change in the refractive index of blood. This change in refractive index results in a shift in the scattering pattern. Quantification of this shift in the scattering pattern enables high-accuracy [Hb] measurement in whole blood sample.

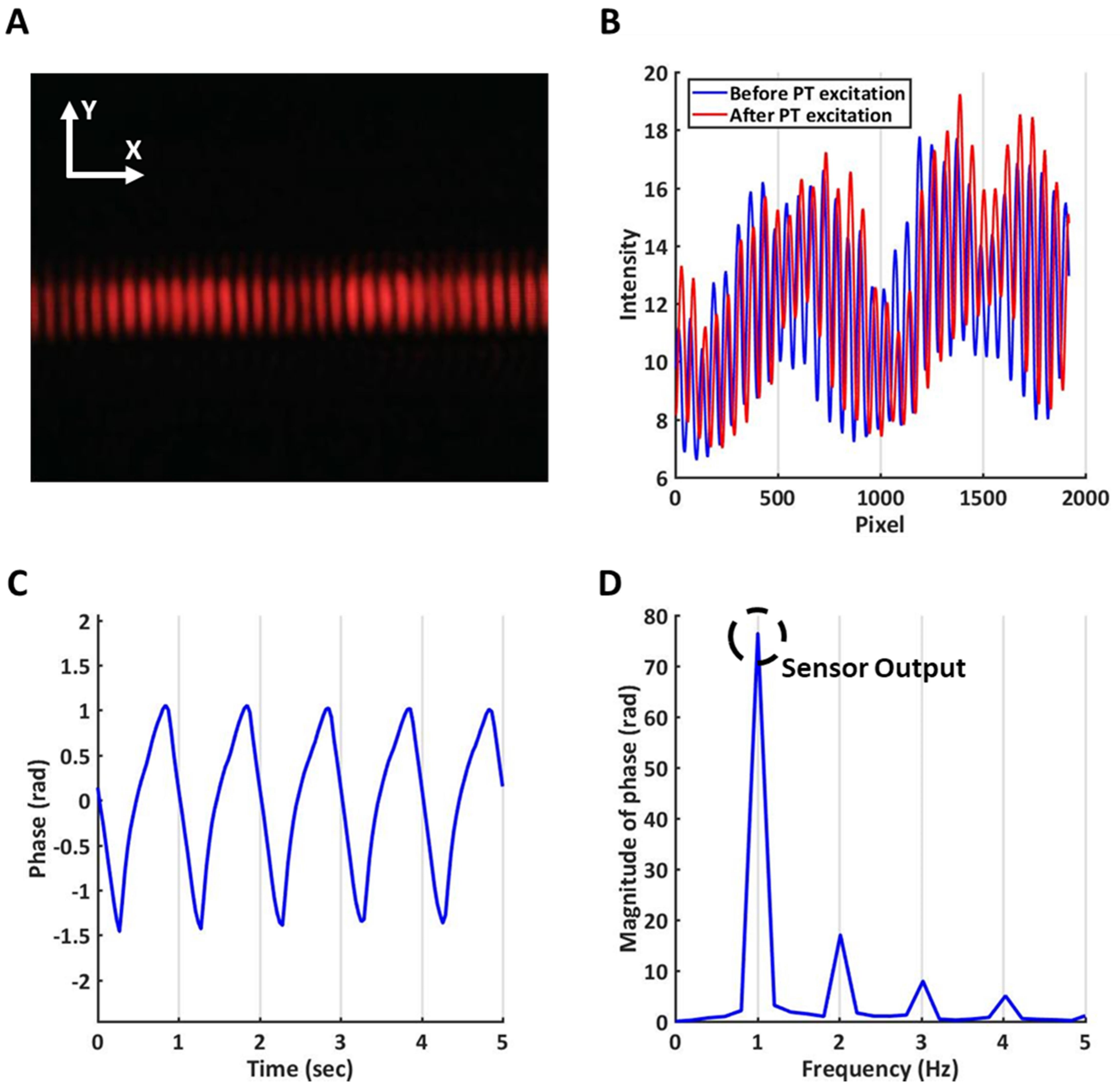


**Fig. S2.** Data processing of m-PTAS sensor. **(A)** A representative image of 650-nm light angular scattering pattern acquired by a CMOS image sensor. **(B)** Arithmetic mean of the pixel values along the vertical (Y) direction before and after PT excitation. **(C)** Phase change of the angular scattering pattern under the 1-Hz modulated PT light illumination. **(D)** Magnitude of the Fourier transform of the phase signal **(C)**. m-PTAS sensor output is the magnitude at the modulation frequency (1Hz).

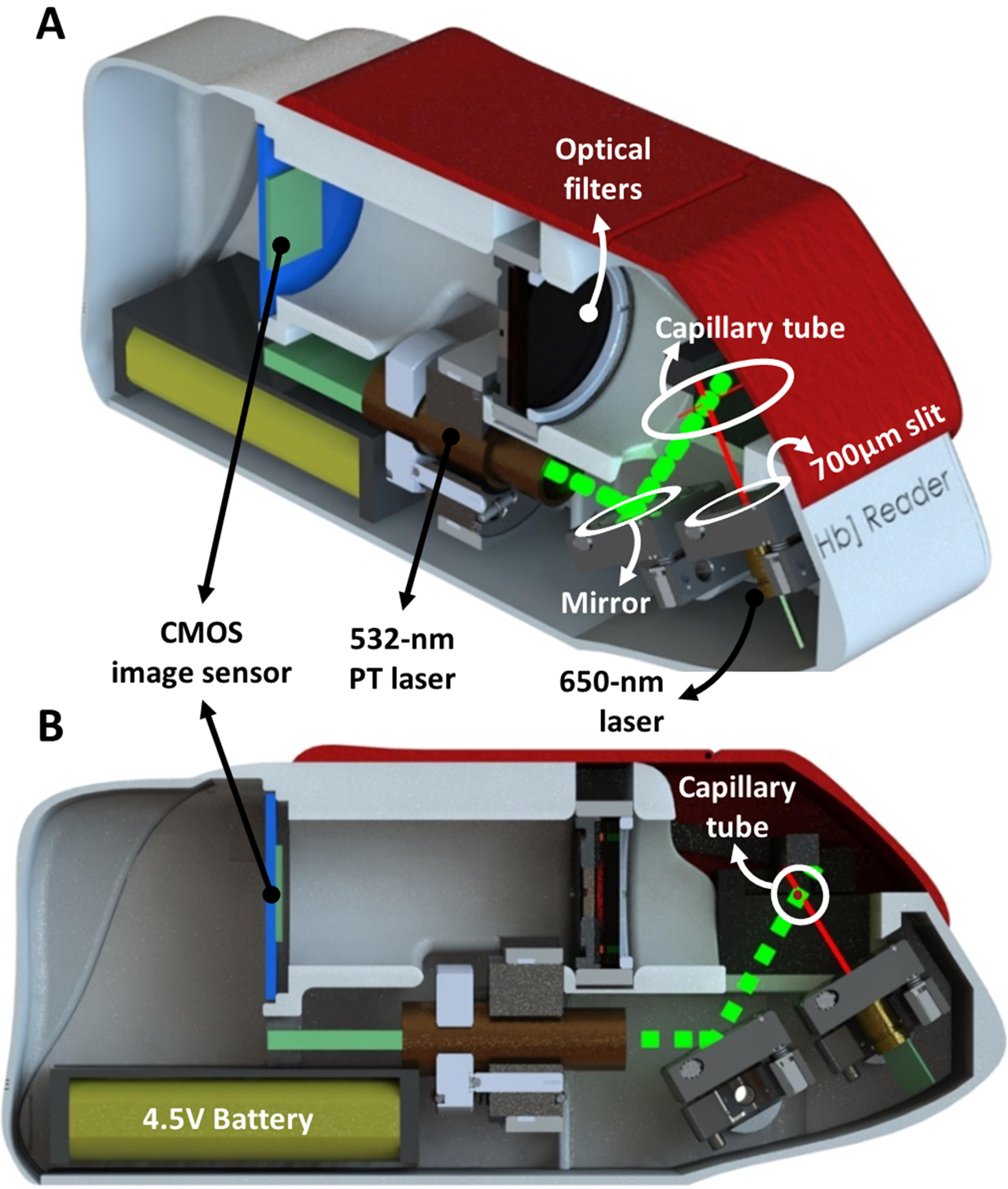


**Fig. S3.** Detailed optical layout of m-PTAS. Cutaway views for m-PTAS device are provided in **(A)** and **(B)** for a better visualization of m-PTAS optical arrangement.

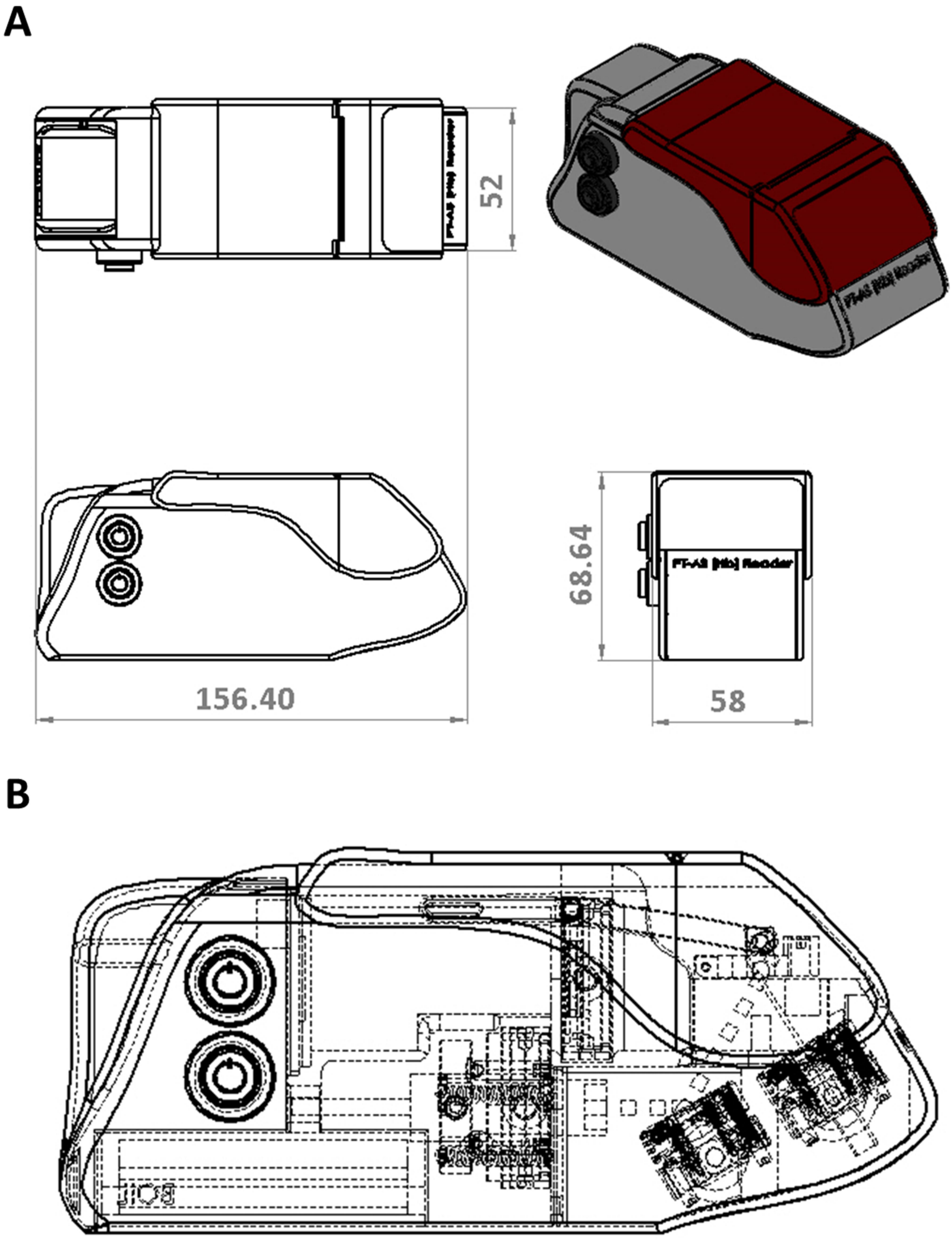


**Fig. S4.** Optomechanical design of m-PTAS sensor. Trimetric projection view **(A)** and assembly drawing **(B)** of m-PTAS sensor.

| Specimen No. | ADVIA 2120i | m-PTAS | |
|---|---|---|---|
| | [Hb] (g/dL) | Sensor Signal (rad) | 95% CI (rad) |
| 1 | 0.1 | 0.8821 | 0.0172 |
| 2 | 1.65 | 22.5732 | 0.9586 |
| 3 | 3.1 | 42.6253 | 1.4936 |
| 4 | 4.8 | 52.2713 | 0.8077 |
| 5 | 5.8 | 54.5785 | 0.7037 |
| 6 | 7.1 | 57.5282 | 0.8721 |
| 7 | 8.0 | 58.1782 | 1.1062 |
| 8 | 9.0 | 60.5900 | 0.9745 |
| 9 | 10.0 | 63.7736 | 1.0732 |
| 10 | 11.0 | 65.6946 | 0.7111 |
| 11 | 12.0 | 68.6837 | 0.8808 |
| 12 | 13.0 | 69.9806 | 0.4179 |
| 13 | 14.0 | 71.9027 | 0.4006 |
| 14 | 15.0 | 72.6859 | 0.7869 |
| 15 | 16.0 | 73.2531 | 1.3245 |
| 16 | 17.8 | 74.8976 | 0.7189 |

**Table S1.** Specimen information for m-PTAS calibration

| Calibration Curve | | |
|---|---|---|
| $$y = A1 + \frac{(A2 - A1) \times \boldsymbol{p}}{\mathbf{1} + \mathbf{10}^{(\boldsymbol{LOGxO1} - \boldsymbol{x}) \times \boldsymbol{h1}}} + \frac{(A2 - A1) \times (1 - \boldsymbol{p})}{\mathbf{1} + \mathbf{10}^{(\boldsymbol{LOGxO2} - \boldsymbol{x}) \times \boldsymbol{h2}}}$$ | | |
| **Parameter** | **Value** | **Standard Error** |
| $A1$ | $-10.8917$ | 2.5384 |
| $A2$ | 75.6260 | 0.5187 |
| $LOGx01$ | 10.3128 | 0.2795 |
| $LOGx02$ | 1.5773 | 0.0824 |
| $h1$ | 0.1878 | 0.0180 |
| $h2$ | 0.4438 | 0.0406 |
| $p$ | 0.2656 | 0.0242 |

**Table S2. Calibration curve.** A Hill equation was employed to fit the m-PTAS calibration measurements to construct a sigmoidal bi dose-response curve.

| Specimen no. | ADVIA 2120i [Hb] (g/dL) | m-PTAS [Hb] (g/dL) | Hb201+ [Hb] (g/dL) | Specimen no. | ADVIA 2120i [Hb] (g/dL) | m-PTAS [Hb] (g/dL) | Hb201+ [Hb] (g/dL) |
|---|---|---|---|---|---|---|---|
| 1 | 5.2 | 5.4 | 5.3 | 126 | 11.7 | 11.9 | 11.8 |
| 2 | 5.9 | 6.0 | 6.0 | 127 | 12.1 | 12.6 | 12.6 |
| 3 | 6.0 | 5.9 | 5.9 | 128 | 12.2 | 12.3 | 12.5 |
| 4 | 6.2 | 6.2 | 6.8 | 129 | 12.4 | 12.6 | 12.9 |
| 5 | 6.5 | 6.6 | 6.7 | 130 | 12.5 | 12.6 | 12.8 |
| 6 | 6.5 | 6.6 | 6.5 | 131 | 12.6 | 13.1 | 13.1 |
| 7 | 6.8 | 5.7 | 6.9 | 132 | 12.8 | 13.5 | 13.4 |
| 8 | 6.9 | 6.7 | 7.1 | 133 | 12.9 | 13.3 | 13.2 |
| 9 | 7.0 | 7.1 | 6.3 | 134 | 13.0 | 13.5 | 13.3 |
| 10 | 7.1 | 7.3 | 7.6 | 135 | 13.2 | 13.4 | 13.5 |
| 11 | 7.2 | 7.4 | 7.3 | 136 | 13.1 | 13.6 | 13.6 |
| 12 | 7.2 | 7.2 | 7.3 | 137 | 13.2 | 13.5 | 13.6 |
| 13 | 7.3 | 7.1 | 7.8 | 138 | 13.3 | 14.1 | 14.0 |
| 14 | 7.4 | 8.2 | 7.6 | 139 | 13.4 | 13.8 | 13.7 |
| 15 | 7.5 | 7.6 | 7.5 | 140 | 13.5 | 13.9 | 13.9 |
| 16 | 7.7 | 7.8 | 7.9 | 141 | 13.6 | 13.4 | 13.5 |
| 17 | 7.9 | 7.9 | 8.1 | 142 | 13.7 | 13.8 | 13.9 |
| 18 | 8.0 | 8.1 | 8.2 | 143 | 13.9 | 14.8 | 14.7 |
| 19 | 8.0 | 8.2 | 8.5 | 144 | 14.1 | 14.9 | 14.8 |
| 20 | 8.5 | 8.5 | 8.0 | 145 | 14.1 | 14.5 | 14.5 |
| 21 | 8.6 | 8.0 | 8.6 | 146 | 14.3 | 14.9 | 14.9 |
| 22 | 8.7 | 8.8 | 8.8 | 147 | 14.2 | 14.6 | 14.7 |
| 23 | 9.0 | 8.5 | 8.2 | 148 | 14.5 | 15.1 | 15.2 |
| 24 | 9.1 | 9.2 | 9.4 | 149 | 14.6 | 15.1 | 15.2 |
| 25 | 9.3 | 9.5 | 9.6 | 150 | 14.8 | 15.2 | 15.3 |
| 26 | 9.8 | 9.8 | 9.6 | 151 | 15.0 | 15.5 | 15.3 |
| 27 | 10.0 | 9.9 | 10.4 | 152 | 15.1 | 15.5 | 15.5 |
| 28 | 10.0 | 9.9 | 10.3 | 153 | 15.2 | 15.6 | 15.6 |
| 29 | 10.0 | 9.9 | 9.3 | 154 | 15.5 | 16.3 | 16.3 |
| 30 | 10.1 | 9.6 | 9.7 | 155 | 15.6 | 15.9 | 16.1 |
| 31 | 10.1 | 10.2 | 10.2 | 156 | 15.7 | 16.4 | 16.3 |
| 32 | 10.3 | 10.3 | 10.3 | 157 | 15.9 | 16.6 | 16.7 |
| 33 | 10.4 | 10.6 | 10.4 | 158 | 16.7 | 17.3 | 17.5 |
| 34 | 11.0 | 11.1 | 11.3 | 159 | 6.7 | 6.8 | 6.8 |
| 35 | 11.0 | 10.6 | 10.0 | 160 | 7.2 | 6.8 | 7.1 |
| 36 | 11.0 | 11.0 | 11.5 | 161 | 7.6 | 7.7 | 7.6 |
| 37 | 11.1 | 11.0 | 11.4 | 162 | 7.8 | 7.8 | 8.0 |
| 38 | 11.2 | 11.4 | 10.5 | 163 | 8.1 | 7.8 | 8.1 |
| 39 | 11.3 | 11.4 | 11.3 | 164 | 8.5 | 8.8 | 8.8 |
| 40 | 11.6 | 11.6 | 11.7 | 165 | 8.6 | 8.8 | 8.9 |
| 41 | 11.9 | 11.6 | 11.3 | 166 | 8.7 | 8.6 | 8.6 |
| 42 | 12.0 | 11.8 | 8.9 | 167 | 9.3 | 9.5 | 9.4 |
| 43 | 12.0 | 12.0 | 11.8 | 168 | 9.4 | 10.2 | 9.5 |
| 44 | 12.0 | 12.0 | 12.6 | 169 | 9.5 | 9.7 | 9.6 |
| 45 | 12.1 | 11.8 | 12.5 | 170 | 9.9 | 10.5 | 10.1 |
| 46 | 12.3 | 12.2 | 12.4 | 171 | 10.0 | 10.1 | 10.0 |

| | | | | | | | |
|---|---|---|---|---|---|---|---|
| 47 | 12.3 | 12.3 | 12.5 | 172 | 10.2 | 10.3 | 10.3 |
| 48 | 12.5 | 12.7 | 12.5 | 173 | 10.3 | 10.7 | 10.6 |
| 49 | 12.9 | 12.0 | 11.7 | 174 | 10.5 | 10.6 | 11.0 |
| 50 | 12.9 | 12.6 | 13.2 | 175 | 10.6 | 10.7 | 10.7 |
| 51 | 13.0 | 12.7 | 13.2 | 176 | 11.0 | 11.0 | 11.1 |
| 52 | 13.0 | 12.8 | 13.0 | 177 | 11.1 | 11.6 | 11.3 |
| 53 | 13.0 | 12.8 | 14.4 | 178 | 11.2 | 11.9 | 11.6 |
| 54 | 13.0 | 12.6 | 13.7 | 179 | 11.3 | 11.8 | 11.5 |
| 55 | 13.1 | 12.1 | 13.2 | 180 | 11.4 | 11.4 | 11.4 |
| 56 | 13.3 | 13.3 | 13.4 | 181 | 11.5 | 11.7 | 11.8 |
| 57 | 14.0 | 13.8 | 14.3 | 182 | 11.7 | 12.2 | 12.1 |
| 58 | 14.0 | 14.2 | 15.0 | 183 | 12.3 | 12.7 | 12.9 |
| 59 | 14.0 | 13.4 | 14.8 | 184 | 12.4 | 12.7 | 12.9 |
| 60 | 14.1 | 14.2 | 14.2 | 185 | 12.7 | 13.2 | 13.1 |
| 61 | 14.2 | 14.1 | 14.2 | 186 | 12.8 | 13.4 | 13.3 |
| 62 | 14.3 | 14.4 | 14.4 | 187 | 13.1 | 13.3 | 13.5 |
| 63 | 14.4 | 14.4 | 14.5 | 188 | 13.2 | 13.5 | 13.3 |
| 64 | 14.5 | 14.7 | 14.4 | 189 | 13.2 | 14.0 | 13.7 |
| 65 | 14.9 | 14.6 | 15.0 | 190 | 13.3 | 13.8 | 13.7 |
| 66 | 15.0 | 14.7 | 14.9 | 191 | 13.4 | 14.1 | 13.9 |
| 67 | 15.0 | 14.9 | 15.5 | 192 | 13.5 | 14.2 | 14.0 |
| 68 | 15.0 | 14.9 | 15.3 | 193 | 13.8 | 14.6 | 14.6 |
| 69 | 15.3 | 15.2 | 15.3 | 194 | 13.9 | 14.3 | 14.4 |
| 70 | 15.3 | 15.4 | 15.4 | 195 | 14.0 | 14.7 | 14.8 |
| 71 | 15.4 | 15.8 | 15.2 | 196 | 14.4 | 15.0 | 15.2 |
| 72 | 15.6 | 15.9 | 15.6 | 197 | 14.5 | 15.3 | 15.2 |
| 73 | 15.9 | 15.3 | 15.2 | 198 | 14.9 | 15.6 | 15.3 |
| 74 | 16.0 | 14.2 | 12.8 | 199 | 15.0 | 15.1 | 15.1 |
| 75 | 16.0 | 16.0 | 15.9 | 200 | 15.2 | 15.3 | 15.2 |
| 76 | 16.1 | 16.0 | 16.2 | 201 | 15.5 | 15.9 | 15.9 |
| 77 | 16.3 | 16.9 | 16.2 | 202 | 15.6 | 15.9 | 16.0 |
| 78 | 16.4 | 16.3 | 16.7 | 203 | 15.7 | 15.9 | 16.1 |
| 79 | 16.4 | 16.4 | 16.5 | 204 | 16.3 | 16.8 | 17.0 |
| 80 | 16.7 | 16.7 | 16.8 | 205 | 5.2 | 5.6 | 5.7 |
| 81 | 16.9 | 17.1 | 17.2 | 206 | 6.6 | 6.5 | 6.8 |
| 82 | 17.0 | 17.3 | 18.0 | 207 | 7.0 | 7.7 | 7.2 |
| 83 | 17.0 | 17.7 | 17.9 | 208 | 7.7 | 8.2 | 7.8 |
| 84 | 17.0 | 16.8 | 17.2 | 209 | 8.0 | 8.5 | 8.3 |
| 85 | 17.1 | 17.5 | 17.4 | 210 | 8.5 | 9.4 | 8.5 |
| 86 | 17.1 | 17.3 | 17.4 | 211 | 9.0 | 9.6 | 9.4 |
| 87 | 17.1 | 17.1 | 17.5 | 212 | 9.5 | 10.2 | 9.8 |
| 88 | 17.4 | 17.6 | 17.7 | 213 | 9.9 | 10.3 | 10.0 |
| 89 | 17.8 | 17.6 | 18.4 | 214 | 10.3 | 11.0 | 10.5 |
| 90 | 17.8 | 18.5 | 18.6 | 215 | 10.7 | 11.6 | 11.1 |
| 91 | 17.8 | 18.3 | 17.2 | 216 | 11.0 | 12.1 | 11.8 |
| 92 | 17.9 | 18.5 | 18.2 | 217 | 11.5 | 12.7 | 12.2 |
| 93 | 17.9 | 17.4 | 17.7 | 218 | 12.0 | 13.1 | 12.5 |
| 94 | 18.1 | 17.8 | 19.4 | 219 | 12.4 | 13.4 | 13.4 |

| | | | | | | | |
|---|---|---|---|---|---|---|---|
| 95 | 18.7 | 18.6 | 19.2 | 220 | 13.5 | 15.2 | 13.6 |
| 96 | 18.8 | 18.7 | 18.0 | 221 | 14.7 | 16.0 | 15.9 |
| 97 | 19.4 | 19.1 | 19.9 | 222 | 14.9 | 16.0 | 16.0 |
| 98 | 19.8 | 19.9 | 20.0 | 223 | 15.4 | 16.3 | 16.2 |
| 99 | 20.6 | 20.9 | 21.1 | 224 | 15.6 | 16.8 | 16.3 |
| 100 | 20.6 | 20.2 | 21.2 | 225 | 15.8 | 17.6 | 17.4 |
| 101 | 12.4 | 13.8 | 13.9 | 226 | 16.4 | 18.8 | 17.8 |
| 102 | 12.8 | 13.1 | 13.1 | 227 | 17.7 | 17.3 | 18.9 |
| 103 | 14.1 | 14.4 | 14.6 | 228 | 5.4 | 5.6 | 5.6 |
| 104 | 14.0 | 15.5 | 15.2 | 229 | 6.9 | 7.0 | 7.0 |
| 105 | 14.6 | 15.8 | 15.6 | 230 | 7.8 | 8.3 | 8.4 |
| 106 | 8.6 | 8.6 | 8.7 | 231 | 8.1 | 8.5 | 8.6 |
| 107 | 12.5 | 13.1 | 13.5 | 232 | 9.2 | 9.1 | 9.4 |
| 108 | 11.9 | 12.5 | 12.2 | 233 | 9.7 | 10.0 | 10.0 |
| 109 | 6.4 | 6.3 | 6.6 | 234 | 10.0 | 10.0 | 10.0 |
| 110 | 7.4 | 7.6 | 7.6 | 235 | 10.5 | 10.7 | 10.9 |
| 111 | 8.0 | 8.1 | 8.1 | 236 | 11.0 | 11.3 | 11.4 |
| 112 | 8.5 | 8.6 | 8.5 | 237 | 11.2 | 11.3 | 11.6 |
| 113 | 8.7 | 8.3 | 8.4 | 238 | 11.6 | 11.8 | 12.1 |
| 114 | 8.8 | 8.8 | 8.8 | 239 | 11.9 | 12.3 | 12.5 |
| 115 | 8.9 | 8.9 | 8.9 | 240 | 12.2 | 12.6 | 12.3 |
| 116 | 9.0 | 8.8 | 9.0 | 241 | 12.7 | 12.9 | 13.4 |
| 117 | 9.3 | 9.1 | 9.2 | 242 | 13.0 | 13.8 | 13.7 |
| 118 | 9.5 | 9.4 | 9.5 | 243 | 13.4 | 14.0 | 14.2 |
| 119 | 9.6 | 9.7 | 9.8 | 244 | 13.7 | 14.5 | 14.7 |
| 120 | 10.2 | 10.1 | 10.2 | 245 | 14.0 | 14.8 | 15.0 |
| 121 | 10.7 | 11.1 | 10.9 | 246 | 14.3 | 15.2 | 15.1 |
| 122 | 10.9 | 10.6 | 11.0 | 247 | 14.5 | 15.4 | 15.3 |
| 123 | 11.0 | 11.1 | 11.4 | 248 | 15.3 | 16.3 | 16.3 |
| 124 | 11.1 | 11.2 | 11.1 | 249 | 15.7 | 16.8 | 16.6 |
| 125 | 11.4 | 11.6 | 11.7 | 250 | 16.5 | 17.1 | 17.4 |

**Table S3.** [Hb] results of blood specimens via m-PTAS, Hb201+ and ADVIA 2120i analyzer

| Specimen No. | ADVIA 2120i [Hb] (g/dL) | m-PTAS [Hb] (g/dL) | Hb201+ [Hb] (g/dL) | Gender | Age | Specimen No. | ADVIA 2120i [Hb] (g/dL) | m-PTAS [Hb] (g/dL) | Hb201+ [Hb] (g/dL) | Gender | Age |
|---|---|---|---|---|---|---|---|---|---|---|---|
| 1 | 6.4 | 6.3 | 6.6 | M | 69 | 74 | 6.7 | 6.8 | 6.8 | F | 31 |
| 2 | 7.4 | 7.6 | 7.6 | M | 47 | 75 | 7.2 | 6.8 | 7.1 | F | 55 |
| 3 | 8.0 | 8.1 | 8.1 | M | 54 | 76 | 7.6 | 7.7 | 7.6 | F | 50 |
| 4 | 8.5 | 8.6 | 8.5 | M | 61 | 77 | 7.8 | 7.8 | 8.0 | F | 64 |
| 5 | 8.7 | 8.3 | 8.4 | M | 64 | 78 | 8.1 | 7.8 | 8.1 | F | 79 |
| 6 | 8.8 | 8.8 | 8.8 | M | 54 | 79 | 8.5 | 8.8 | 8.8 | F | 45 |
| 7 | 8.9 | 8.9 | 8.9 | M | 40 | 80 | 8.6 | 8.8 | 8.9 | F | 15 |
| 8 | 9.0 | 8.8 | 9.0 | M | 59 | 81 | 8.7 | 8.6 | 8.6 | F | 37 |
| 9 | 9.3 | 9.1 | 9.2 | M | 87 | 82 | 9.3 | 9.5 | 9.4 | F | 35 |
| 10 | 9.5 | 9.4 | 9.5 | M | 64 | 83 | 9.4 | 10.2 | 9.5 | F | 83 |
| 11 | 9.6 | 9.7 | 9.8 | M | 45 | 84 | 9.5 | 9.7 | 9.6 | F | 45 |
| 12 | 10.2 | 10.1 | 10.2 | M | 80 | 85 | 9.9 | 10.5 | 10.1 | F | 36 |
| 13 | 10.7 | 11.1 | 10.9 | M | 36 | 86 | 10.0 | 10.1 | 10.0 | F | 69 |
| 14 | 10.9 | 10.6 | 11.0 | M | 57 | 87 | 10.2 | 10.3 | 10.3 | F | 33 |
| 15 | 11.0 | 11.1 | 11.4 | M | 64 | 88 | 10.3 | 10.7 | 10.6 | F | 59 |
| 16 | 11.1 | 11.2 | 11.1 | M | 79 | 89 | 10.5 | 10.6 | 11.0 | F | 58 |
| 17 | 11.4 | 11.6 | 11.7 | M | 78 | 90 | 10.6 | 10.7 | 10.7 | F | 64 |
| 18 | 11.7 | 11.9 | 11.8 | M | 14 | 91 | 11.0 | 11.0 | 11.1 | F | 74 |
| 19 | 12.1 | 12.6 | 12.6 | M | 52 | 92 | 11.1 | 11.6 | 11.3 | F | 64 |
| 20 | 12.2 | 12.3 | 12.5 | M | 64 | 93 | 11.2 | 11.9 | 11.6 | F | 66 |
| 21 | 12.4 | 12.6 | 12.9 | M | 88 | 94 | 11.3 | 11.8 | 11.5 | F | 21 |
| 22 | 12.5 | 12.6 | 12.8 | M | 72 | 95 | 11.4 | 11.4 | 11.4 | F | 83 |
| 23 | 12.6 | 13.1 | 13.1 | M | 62 | 96 | 11.5 | 11.7 | 11.8 | F | 85 |
| 24 | 12.8 | 13.5 | 13.4 | M | 37 | 97 | 11.7 | 12.2 | 12.1 | F | 79 |
| 25 | 12.9 | 13.3 | 13.2 | M | 76 | 98 | 12.3 | 12.7 | 12.9 | F | 66 |
| 26 | 13.0 | 13.5 | 13.3 | M | 76 | 99 | 12.4 | 12.7 | 12.9 | F | 64 |
| 27 | 13.2 | 13.4 | 13.5 | M | 65 | 100 | 12.7 | 13.2 | 13.1 | F | 66 |
| 28 | 13.1 | 13.6 | 13.6 | M | 53 | 101 | 12.8 | 13.4 | 13.3 | F | 45 |
| 29 | 13.2 | 13.5 | 13.6 | M | 80 | 102 | 13.1 | 13.3 | 13.5 | F | 72 |
| 30 | 13.3 | 14.1 | 14.0 | M | 44 | 103 | 13.2 | 13.5 | 13.3 | F | 55 |
| 31 | 13.4 | 13.8 | 13.7 | M | 26 | 104 | 13.2 | 14.0 | 13.7 | F | 67 |
| 32 | 13.5 | 13.9 | 13.9 | M | 78 | 105 | 13.3 | 13.8 | 13.7 | F | 43 |
| 33 | 13.6 | 13.4 | 13.5 | M | 70 | 106 | 13.4 | 14.1 | 13.9 | F | 12 |
| 34 | 13.7 | 13.8 | 13.9 | M | 54 | 107 | 13.5 | 14.2 | 14.0 | F | 26 |
| 35 | 13.9 | 14.8 | 14.7 | M | 42 | 108 | 13.8 | 14.6 | 14.6 | F | 65 |
| 36 | 14.1 | 14.9 | 14.8 | M | 34 | 109 | 13.9 | 14.3 | 14.4 | F | 33 |
| 37 | 14.1 | 14.5 | 14.5 | M | 24 | 110 | 14.0 | 14.7 | 14.8 | F | 68 |
| 38 | 14.3 | 14.9 | 14.9 | M | 29 | 111 | 14.4 | 15.0 | 15.2 | F | 43 |
| 39 | 14.2 | 14.6 | 14.7 | M | 59 | 112 | 14.5 | 15.3 | 15.2 | F | 55 |
| 40 | 14.5 | 15.1 | 15.2 | M | 36 | 113 | 14.9 | 15.6 | 15.3 | F | 31 |
| 41 | 14.6 | 15.1 | 15.2 | M | 26 | 114 | 15.0 | 15.1 | 15.1 | F | 28 |
| 42 | 14.8 | 15.2 | 15.3 | M | 14 | 115 | 15.2 | 15.3 | 15.2 | F | 42 |
| 43 | 15.0 | 15.5 | 15.3 | M | 38 | 116 | 15.5 | 15.9 | 15.9 | F | 54 |
| 44 | 15.1 | 15.5 | 15.5 | M | 24 | 117 | 15.6 | 15.9 | 16.0 | F | 60 |
| 45 | 15.2 | 15.6 | 15.6 | M | 29 | 118 | 15.7 | 15.9 | 16.1 | F | 14 |
| 46 | 15.5 | 16.3 | 16.3 | M | 39 | 119 | 16.3 | 16.8 | 17.0 | F | 72 |

| | | | | | | | | | | | |
|---|---|---|---|---|---|---|---|---|---|---|---|
| 47 | 15.6 | 15.9 | 16.1 | M | 60 | 120 | 5.4 | 5.6 | 5.6 | F | 76 |
| 48 | 15.7 | 16.4 | 16.3 | M | 23 | 121 | 6.9 | 7.0 | 7.0 | F | 67 |
| 49 | 15.9 | 16.6 | 16.7 | M | 46 | 122 | 7.8 | 8.3 | 8.4 | F | 54 |
| 50 | 16.7 | 17.3 | 17.5 | M | 38 | 123 | 8.1 | 8.5 | 8.6 | F | 47 |
| 51 | 5.2 | 5.6 | 5.7 | M | 89 | 124 | 9.2 | 9.1 | 9.4 | F | 72 |
| 52 | 6.6 | 6.5 | 6.8 | M | 66 | 125 | 9.7 | 10.0 | 10.0 | F | 79 |
| 53 | 7.0 | 7.7 | 7.2 | M | 49 | 126 | 10.0 | 10.0 | 10.0 | F | 83 |
| 54 | 7.7 | 8.2 | 7.8 | M | 22 | 127 | 10.5 | 10.7 | 10.9 | F | 41 |
| 55 | 8.0 | 8.5 | 8.3 | M | 51 | 128 | 11.0 | 11.3 | 11.4 | F | 27 |
| 56 | 8.5 | 9.4 | 8.5 | M | 75 | 129 | 11.2 | 11.3 | 11.6 | F | 72 |
| 57 | 9.0 | 9.6 | 9.4 | M | 66 | 130 | 11.6 | 11.8 | 12.1 | F | 84 |
| 58 | 9.5 | 10.2 | 9.8 | M | 85 | 131 | 11.9 | 12.3 | 12.5 | F | 50 |
| 59 | 9.9 | 10.3 | 10.0 | M | 66 | 132 | 12.2 | 12.6 | 12.3 | F | 62 |
| 60 | 10.3 | 11.0 | 10.5 | M | 72 | 133 | 12.7 | 12.9 | 13.4 | F | 71 |
| 61 | 10.7 | 11.6 | 11.1 | M | 60 | 134 | 13.0 | 13.8 | 13.7 | F | 71 |
| 62 | 11.0 | 12.1 | 11.8 | M | 76 | 135 | 13.4 | 14.0 | 14.2 | F | 33 |
| 63 | 11.5 | 12.7 | 12.2 | M | 69 | 136 | 13.7 | 14.5 | 14.7 | F | 20 |
| 64 | 12.0 | 13.1 | 12.5 | M | 65 | 137 | 14.0 | 14.8 | 15.0 | F | 15 |
| 65 | 12.4 | 13.4 | 13.4 | M | 33 | 138 | 14.3 | 15.2 | 15.1 | F | 66 |
| 66 | 13.5 | 15.2 | 13.6 | M | 54 | 139 | 14.5 | 15.4 | 15.3 | F | 18 |
| 67 | 14.7 | 16.0 | 15.9 | M | 42 | 140 | 15.3 | 16.3 | 16.3 | F | 59 |
| 68 | 14.9 | 16.0 | 16.0 | M | 33 | 141 | 15.7 | 16.8 | 16.6 | F | 66 |
| 69 | 15.4 | 16.3 | 16.2 | M | 13 | 142 | 16.5 | 17.1 | 17.4 | F | 68 |
| 70 | 15.6 | 16.8 | 16.3 | M | 83 | | | | | | |
| 71 | 15.8 | 17.6 | 17.4 | M | 60 | | | | | | |
| 72 | 16.4 | 18.8 | 17.8 | M | 51 | | | | | | |
| 73 | 17.7 | 17.3 | 18.9 | M | 17 | | | | | | |

**Table S4.** [Hb] results of blood specimens used for anemia classification. The [Hb] results were acquired with m-PTAS, Hb201+, and ADVIA 2120i analyzer

**(A)**

| Anemia classification: male | | ADVIA 2120i | | Total |
|---|---|---|---|---|
| | | Positive | Negative | |
| m-PTAS | Positive | 35 | 0 | 35 |
| | Negative | 5 | 33 | 38 |
| Total | | 40 | 33 | 73 |
| Analysis | | **87.50%** | **100.00%** | **93.15%** |
| | | Sensitivity | Specificity | Accuracy |

**(B)**

| Anemia classification: female | | ADVIA 2120i | | Total |
|---|---|---|---|---|
| | | Positive | Negative | |
| m-PTAS | Positive | 34 | 0 | 34 |
| | Negative | 2 | 33 | 35 |
| Total | | 36 | 33 | 69 |
| Analysis | | **94.44%** | **100.00%** | **97.10%** |
| | | Sensitivity | Specificity | Accuracy |

**(C)**

| Anemia classification : male | | ADVIA 2120i | | Total |
|---|---|---|---|---|
| | | Positive | Negative | |
| Hb201+ | Positive | 36 | 0 | 36 |
| | Negative | 4 | 33 | 37 |
| Total | | 40 | 33 | 73 |
| Analysis | | **90.00%** | **100.00%** | **94.52%** |
| | | Sensitivity | Specificity | Accuracy |

**(D)**

| Anemia classification: female | | ADVIA 2120i | | Total |
|---|---|---|---|---|
| | | Positive | Negative | |
| Hb201+ | Positive | 33 | 0 | 33 |
| | Negative | 3 | 33 | 36 |
| Total | | 36 | 33 | 69 |
| Analysis | | **91.67%** | **100.00%** | **95.65%** |
| | | Sensitivity | Specificity | Accuracy |

**Table S5.** Contingency table comparing two hemoglobinometers (m-PTAS and Hb201+) and ADVIA 2120i under clinical cut-offs for anemia (13 g/dL for males and 12 g/dL for females). Anemic patients were confirmed by ADVIA 2120i analyzer. Positive refers to patients diagnosed with anemia and negative refers to patients not diagnosed with anemia using the two hemoglobinometers.

| | Components | Quantity | Cost | Manufacturer | Country |
|---|---|---|---|---|---|
| **Light Source** | 532-nm PT laser | 1 | $50.94 | Lilly Electronics | China |
| | 650-nm laser | 1 | $1.07 | Besram Technology Inc. | China |
| **Image Sensor** | CMOS image sensor | 1 | $34.78 | Ailipu Technology Co., Ltd | China |
| **Optical Filter** | IR cut filter | 1 | $38.50 | Edmund Optics | USA |
| | Neutral density filter | 1 | $34.41 | Thorlabs | USA |
| | Long-pass Filter | 1 | $37.30 | Thorlabs | USA |
| **Electronic component** | Arduino-nano | 1 | $2.86 | Jiangsu Yuheng Co., Ltd | China |
| | Pushbutton power switch | 1 | $4.75 | Pololu Corporation | USA |
| | **Total** | | **$204.61** | | |

**Table S6.** Bill of materials for m-PTAS implementation. Note that the cost can be further reduced with smaller image sensor and optical elements.

| Specimen No. | m-PTAS [Hb] (g/dL) | Computation Time | Specimen No. | m-PTAS [Hb] (g/dL) | Computation Time |
|---|---|---|---|---|---|
| 1 | 5.3701 | 2.19 | 51 | 12.6788 | 2.20 |
| 2 | 6.0397 | 2.39 | 52 | 12.8259 | 2.31 |
| 3 | 5.9122 | 2.31 | 53 | 12.7901 | 2.32 |
| 4 | 6.1789 | 2.44 | 54 | 12.6004 | 2.20 |
| 5 | 6.6191 | 2.43 | 55 | 12.08 | 2.29 |
| 6 | 6.5718 | 2.47 | 56 | 13.3033 | 2.31 |
| 7 | 5.6761 | 2.44 | 57 | 13.8141 | 2.24 |
| 8 | 6.6866 | 2.43 | 58 | 14.2347 | 2.29 |
| 9 | 7.1344 | 2.45 | 59 | 13.4474 | 2.20 |
| 10 | 7.3111 | 2.42 | 60 | 14.1779 | 2.25 |
| 11 | 7.4106 | 2.31 | 61 | 14.1313 | 2.32 |
| 12 | 7.2363 | 2.30 | 62 | 14.437 | 2.36 |
| 13 | 7.141 | 2.33 | 63 | 14.4485 | 2.30 |
| 14 | 8.1669 | 2.21 | 64 | 14.6857 | 2.30 |
| 15 | 7.5825 | 2.27 | 65 | 14.6029 | 2.29 |
| 16 | 7.8372 | 2.42 | 66 | 14.7024 | 2.29 |
| 17 | 7.8825 | 2.31 | 67 | 14.8602 | 2.24 |
| 18 | 8.1476 | 2.21 | 68 | 14.9374 | 2.30 |
| 19 | 8.1593 | 2.30 | 69 | 15.2381 | 2.30 |
| 20 | 8.4545 | 2.22 | 70 | 15.3962 | 2.20 |
| 21 | 7.957 | 2.31 | 71 | 15.7851 | 2.22 |
| 22 | 8.7563 | 2.24 | 72 | 15.8555 | 2.29 |
| 23 | 8.4856 | 2.21 | 73 | 15.2703 | 2.21 |
| 24 | 9.2411 | 2.21 | 74 | 14.24 | 2.29 |
| 25 | 9.4684 | 2.21 | 75 | 15.9998 | 2.30 |
| 26 | 9.8233 | 2.30 | 76 | 16.0272 | 2.30 |
| 27 | 9.905 | 2.30 | 77 | 16.8639 | 2.24 |
| 28 | 9.9096 | 2.23 | 78 | 16.2982 | 2.25 |
| 29 | 9.8901 | 2.30 | 79 | 16.3816 | 2.30 |
| 30 | 9.5829 | 2.23 | 80 | 16.7452 | 2.29 |
| 31 | 10.1895 | 2.35 | 81 | 17.0797 | 2.30 |
| 32 | 10.2906 | 2.31 | 82 | 17.3101 | 2.20 |
| 33 | 10.6462 | 2.30 | 83 | 17.7049 | 2.30 |
| 34 | 11.1122 | 2.45 | 84 | 16.8189 | 2.27 |
| 35 | 10.5658 | 2.29 | 85 | 17.4501 | 2.31 |
| 36 | 10.9557 | 2.28 | 86 | 17.2885 | 2.38 |
| 37 | 10.9697 | 2.29 | 87 | 17.1292 | 2.30 |
| 38 | 11.3793 | 2.21 | 88 | 17.5654 | 2.28 |
| 39 | 11.357 | 2.30 | 89 | 17.6498 | 2.20 |
| 40 | 11.5965 | 2.36 | 90 | 18.537 | 2.29 |
| 41 | 11.5637 | 2.30 | 91 | 18.272 | 2.24 |
| 42 | 11.7965 | 2.33 | 92 | 18.4902 | 2.31 |
| 43 | 11.9981 | 2.30 | 93 | 17.4393 | 2.22 |
| 44 | 11.9524 | 2.24 | 94 | 17.8428 | 2.29 |
| 45 | 11.832 | 2.29 | 95 | 18.5558 | 2.22 |
| 46 | 12.2322 | 2.33 | 96 | 18.665 | 2.28 |
| 47 | 12.3027 | 2.32 | 97 | 19.1085 | 2.25 |
| 48 | 12.7243 | 2.30 | 98 | 19.8945 | 2.20 |
| 49 | 12.0301 | 2.32 | 99 | 20.8597 | 2.30 |
| 50 | 12.6497 | 2.22 | 100 | 20.2126 | 2.30 |

**Table S7.** m-PTAS sensor computation time. We measured computation time required for smartphone application, “meaHb” for 100 blood specimens. On an average, 2.29 s with a standard deviation of 0.06 s was evaluated as the computation time.

**Movie S1.** Videoclip of smartphone-based m-PTAS operation

**Movie S2.** m-PTAS images of probe light angular scattering pattern. The pattern shifts under the modulated PT light illumination.
